# Modified Protocol for Establishment of Intracranial Arterial Dolichoectasia Model by Injection of Elastase Into Cerebellomedullary Cistern in Mice

**DOI:** 10.3389/fneur.2022.860541

**Published:** 2022-04-18

**Authors:** Fei Xiang Liu, Yu Ge Niu, Dao Pei Zhang, Huai Liang Zhang, Zhen Qiang Zhang, Rui Qin Sun, Yun Ke Zhang

**Affiliations:** ^1^The First Affiliated Hospital of Henan University of Chinese Medicine, Zhengzhou, China; ^2^Henan Vertigo Disease Diagnosis and Treatment Center, Zhengzhou, China; ^3^Institute of Vertigo Disease, Henan University of Chinese Medicine, Zhengzhou, China; ^4^College of Traditional Chinese Medicine, Henan University of Traditional Chinese Medicine Academy, Zhengzhou, China; ^5^Research and Experiment Center, Henan University of Chinese Medicine, Zhengzhou, China; ^6^School of Rehabilitation Medicine, Henan University of Chinese Medicine, Zhengzhou, China

**Keywords:** intracranial arterial dolichoectasia, elastase, cerebellomedullary cistern, stroke, matrix metalloproteinase-9

## Abstract

**Background and Purpose:**

This study aimed to construct an animal model of intracranial arterial dolichoectasia (IADE) applying the modified modeling protocol.

**Materials and Methods:**

Twenty five milliunits elastase and inactivated elastase were, respectively, injected into the cerebellomedullary cistern of 60 C57/BL6 mice which were divided into experimental group (EG, *n* = 30) and control group (CG, *n* = 30) by using a computer-based random order generator. The modified modeling protocol clarified these aspects including brain three-dimensional parameters of mouse head fixation, angle of head inclination, fixed position of taper ear, needle holding technique, needle entry depth, prevention of liquid drug back flow, and storage conditions of elastase. And it was observed for the following parts such as mortality, inflammatory factors, craniocerebral arteries scanning, vascular tortuosity index, artery diameter, pathology of the cerebrovascular.

**Results:**

Within differently surveyed stage, the total mortality of mice in EG was 20%. ELISA illustrated that the levels of matrix metalloproteinase-9 (MMP-9) and tumor necrosis factor α (TNF-α) in peripheral blood were increased significantly after modeling. Angiography indicated that 100% of IADE in EG were observed and the diameter and tortuosity index of the basilar artery were significantly increased (*P* < 0.01). EVG histological processing and staining showed the disrupted internal elastic lamina, the atrophied muscle layer, and the hyalinized connective tissue of the basilar artery with the vascular wall tunica media in EG. Micro-computed tomography reported that the craniocerebral arteries of the mice in EG were outstandingly elongated, tortuous, and dilated.

**Conclusion:**

The modified modeling protocol can reduce the mortality, improve the success rate, and provide a stable animal model for IADE.

## Introduction

Intracranial arterial dolichoectasia (IADE) is an intractable cerebrovascular disease characterized by the elongation, dilation, and tortuosity of intracranial arteries (1). The main pathological changes are the damage of the internal elastic layer, atrophy of the vascular muscles, and hyaline degeneration of connective tissue (1). IADE is associated with lacunar infarction, brain microbleeds, enlarged perivascular space, and other cerebral small vessel disease, which could cause the compression of brainstem, hydrocephalus, intracranial hemorrhage, etc, seriously affect patient's life safety and bring a heavy medical burden to the society (2). Recent clinical studies have pointed out that its pathogeneses may include: ① Abnormal vascular reconstruction and vascular wall structure were caused by the imbalance of matrix metalloprotein and anti-protease activities (3). ② There are two-way flows in the dilated artery segment, and the reverse blood flow slows down the velocity of forward blood flow, which can easily form thrombus and produce cerebral embolism (4). ③ The distortion and deformation of blood vessels lead to the occlusion of perforating arteries and the occurrence of cerebral small vessel disease (5). ④ Ectopic blood vessels compress surrounding structures, including brainstem and cranial nerves (6). The compression of the third ventricle leads to the obstruction of the flow of cerebrospinal fluid, resulting in the “water hammer” effect (7). The rupture of elastic fiber of endarterium, thinning of tunica media, as well as the chronic local shearing force lead to the angiorrhexis, resulting in intracerebral hemorrhage and subarachnoid hemorrhage (8). However, the pathophysiological mechanism of IADE is still not clear, and there are no accurate and effective means of prevention and treatment. Therefore, it is necessary to explore the animal models with IADE characteristics.

In 2008, researchers established an IADE model by ligating the rabbit arteria carotis communis to simulate the extension and bending of the basilar artery induced by hemodynamic changes (9). Since 2017, researchers in Mayo Clinic have successfully developed an IADE animal model by injecting elastase into the cerebellomedullary cistern. In this research, a series of studies were conducted, including the exploration of modeling methods, animal strains, drug doses, and modeling time. The results showed that C57/BL6 mice were the best strains, the best concentration of elastase was 25 milliunits (mu), as well as the successful modeling time was 14 d (10, 11). These studies provide the detailed basic data to explore the pathology of IADE. Nevertheless, the current mortality rate of this model is as high as 30%, and the success rate of elongation and dilatation of intracranial arteries after modeling is only 60–67% (10, 11). The present study aims to reduce the mortality of the IADE model constructed by injecting elastase into the cerebellomedullary cistern of mice, which can improve the success rate of the model and establish a stable and reliable animal model system. Combined with our previous experience and lessons, this study optimized the modeling method and formed a modified modeling protocol. Based on the following aspects including the fixation parameters of stereotaxic apparatus and angles, needle entry manipulation, needle entry depth, and mouse brain stereotactic positioning, the key points of establishing this model were discussed in detail. The modified modeling protocol was applied to facilitate scientific researches and explore the pathophysiological mechanisms of IADE.

## Materials

### Instruments and Reagents

The stereotaxic apparatus (Item No.: 51650) was provided by Shanghai Yuyan Technology Co., Ltd., The 10 μL micro-nano syringe was purchased from Hamilton company (Romania). Enzyme-linked immune detector (EnSpire) was provided by Perkin Elmer Instrument Co., Ltd., USA. Micro-CT [VENUS (VNC-102)] was purchased from Jiangsu, Kunshan Pingsheng Technology Co., LTD., China.

The vascular contrast agents of FlowTech MICROFIL (Batch No. BOX834) was provided by FlowTech, Inc. Elastase was purchased from Sigma (Batch No. E57758). Isoflurane was provided by Aladdin^®^ (Batch No. 26675-46-7). EVG Staining Kit was purchased from Service (Batch No. G1042). Matrix metalloproteinase-9 (MMP-9) and tumor necrosis factor α (TNF-α) ELISA kits were provided by Shanghai Enzyme-linked Biotechnology Co., Ltd. (Batch Nos. ml058617-1, ml002095-1).

### Experiment Animals

Sixty 2-month-old SPF C57/BL6 mice weighed 25 ± 2 g were provided by SpefoBio-Company with the certificate number of SCXK (Beijing) 2019-0010. Mice were kept in the Animal Experimental Center of Henan University of Chinese Medicine (5 per cage), with free access to water and food, at the temperature of 23.1 ± 2.5°C and the humidity of 55.3 ± 2.5%. This modeling protocol and operation procedures were approved by the Ethics Committee of Henan University of Chinese Medicine (Ethics Number: DWLL202107012).

### Grouping and Administration

Mice were randomly allocated to the experimental group (EG, *n* = 30) and the control group (CG, *n* = 30) using a computer-based random order generator. EG was given 2.5 μL of elastase with a concentration of 10 mu/μL, while CG was given the same volume of inactivated elastase.

Ten mu/μL of elastase was prepared with 5 mg of elastase (7 units/mg) dissolved in 3.5 mL of sterile PBS. Then it was packaged and stored at −20°C. The solution shall be kept at 0°C to protect elastase against inactivation when it is used.

Ten mu/μL of inactivated elastase was also prepared. Elastase with the concentration of 10 mu/μL was heated at 100°C in thermostat water bath for 10 min until it was completely inactivated.

## Methods

### Modeling

The modeling process referred to the method of Dai et al. (10, 11). This study improved the fixed position of the mouse head, parameters of ear tapers, the angle range between the neck and spine, the method of holding needle, the depth of needle insertion, as well as the method of preventing medicine liquid backflow.

**Figure 1:**
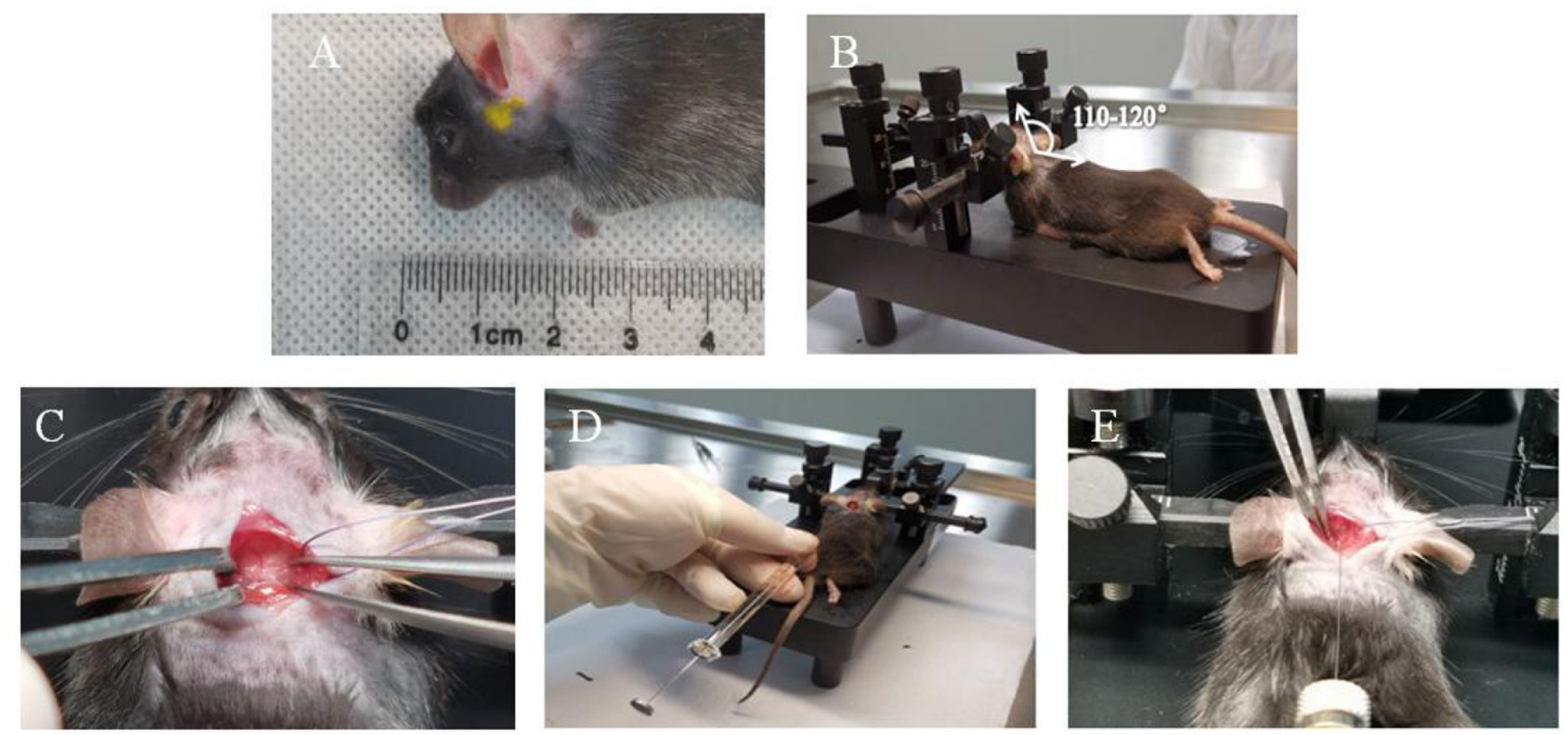
Key operating steps of mouse modeling. **(A)** The fixed position of the mouse ear cone is the preauricular temporal bone. **(B)** The angle between the mouse neck and the body is 110–120°C. **(C)** Dura mater. **(D)** Needle holding position. **(E)** Horizontal needle.

(1) Mice were acclimated to the laboratory environment for 1 week. It needed 8 h of preoperative fasting food and water. The isoflurane inhalations flowed at the rate of 1 mL/min which could keep animals under anesthesia.
(2) The shaver was used to shave the mouse hair of neck in preoperative skin preparation. Mouse was fixed on the stereotaxic apparatus with the following parameters: ① The incisors of mice were fixed to the maxillary fixator of brain locator with a height of 14–15 mm. ② Both ear tapers were fixed to the preauricular temporary bone with a width of 5–6 mm. ③ The height of the left and right ear tapers was adjusted by 15–16 mm and kept the same level. ④ Under the fixed conditions of these parameters, the head of the mouse was parallel to the plane and was 110–120° to the body (Figure 1).
(3) Conventional surgery area was disinfected with iodophor three times, 75% alcohol for deiodination two times, and sterile surgical towels single was paved.
(4) An incision about 1 cm in length was made along the inferior nuchal line of occipital, and then the muscular layer was cut along the linea alba cervicalis. Afterward, the muscle attached to the foramen magnum of occipital bone was separated using dissecting forceps to fully expose the dura mater spinalis.
(5) The microsyringe was held with the left thumb, index finger and middle finger. The left ring finger and little finger depended on the stereotaxic apparatus to prevent the hand from shaking and inserting too deep or inserting wrong direction.
(6) The intersection of midline of skull with the horizontal line of foramen magnum was taken as the insertion point. Microsyringe was slowly penetrated into the foramen magnum with a depth about 1 mm. Then, the insertion was immediately stopped when a slight give in pressure was felt. This step was of vital importance which related to the life of mice to a great extent.
(7) It showed that the position of microsyringe entered cerebellomedullary cistern was correct when cerebrospinal fluid was drew out. The position was wrong, if the cerebrospinal fluid could not be drew out. Microsyringe should be pulled out when attempted a few times. This step was largely related to whether the elastase could be injected into the target area correctly.
(8) 2.5 μL solution of 25 mu elastase was slowly injected into the cerebellomedullary cistern within 10 s. Then, it should immediately press the pinhole with a cotton swab when pulling out the microsyringe, to prevent the elastase from flowing out. This step was related to whether the entire drug dose could stay in brain.
(9) 4-0 absorbable surgical thread was used to sew up the muscular layer, fascia layer, as well as cortex in turn. The operative incision was disinfected with the endeavor and externally applied with 40 thousand IU of penicillin G sodium per mouse.
(10) In CG, 25 mu inactivated elastase was injected into the cerebellomedullary cistern. After operation, it needed to maintain the temperature at 37°C to keep the mice warm until they woke up. The operative incision was checked every day to protect animals from biting each other which might cause unnecessary death.

### Survival Record

After operation, the survival status and mortality of mice were recorded in 24 h, 1–3 d, 4–7 d, and 8–14 d.

### Angiography of FlowTech Microfil

After 2 weeks of modeling, 19 mice from EG and 22 mice from CG were randomly selected for angiography using FlowTech MICROFIL contrast medium. The MV-Diluent, MV-122, and MV-Curing agent of FlowTech MICROFIL were blended with the proportion of 5 mL/4 mL/5% of the total volume, respectively, and used within 3 min. The pleura was opened to expose the heart, under anesthetic with an intraperitoneal injection of sodium pentobarbital (30 mg/kg). The cardio-perfusion technique, 4% polyformaldehyde after normal saline, was used in heart beating mice. Then, 3 ml of FlowTech MICROFIL was used for cerebral angiography within 3 min. The skull was cut off to fully expose the brain tissue. It was carefully separated from the cranial cavity along with the cerebral vascular network under the anatomical microscope after mice were fixed in a refrigerator at 4°C for 24 h. All the brain tissues were fixed with 10% formaldehyde buffer, and kept in a constant-temperature room for at least 24 h before imaging.

### Morphological Measurement

Q-capture Pro 7 software was used to capture images and calculate the vascular tortuosity index (TI) and the percentage increase in arterial diameter. The maximum arteries diameter of basilar artery, bilateral anterior cerebral arteries and bilateral posterior communicating arteries were measured by Image-Pro Plus software. The percentage increase in arterial diameter {[(actual diameter measurement – sham mean diameter)/sham mean diameter] × 100%} was used to compare the diameters among groups. Dilation was defined as a percentage increase in diameter ≥25%. In this study, the previously verified bending index was used to calculate the TI of vessels that reported by Dai et al. (10, 11). The TI was calculated as follows: (actual length of blood vessel/straight length of blood vessel −1) × 100. Total value micro-image was obtained using image-Pro Plus software and TI > 10 was defined as tortuosity.

### Detection of Serum Inflammatory Factor

After 2 weeks of modeling, the whole blood samples of mice were collected and naturally solidified at room temperature for 0.5 h. Samples were centrifuged at 2,500 rpm for 20 min, and the supernatants were collected in centrifuge tubes. The levels of MMP-9 and TNF-α in serum were measured following the instructions. Then, serum inflammatory factors were assessed at an absorbance value of 450 nm using enzyme-linked immune detector.

### Histological Treatment and Staining of EVG

Five mice from each group were randomly selected. The epencephals containing basilar artery vessels of mice were collected and fixed in 4% paraformaldehyde. The paraffin-embedded basilar artery tissues were sectioned into 4-μm-thick specimens for EVG staining. The stained basilar artery vessels were observed and photographed under fluorescent microscopy with the imaging system of NIKON digital sight DS-FI2 (Nikon Eclipse ci, Tokyo, Japan).

### Micro-CT

The cerebral arteries of mice that had been accepted Angiography of FlowTech MICROFIL were measured, and using Micro-CT with the parameters as follows, tube voltage: 90 VK, tube current: 0.06 mA, reconstruction type: FDK, transverse FOV: 30 mm, axial FOV 10 mm, and pixel resolution: 0.015 × 0.015 × 0.025 mm. After scanned, the vascular images were reconstructed using Skyscan NRecon software. Then, Data viewer was used for intensity-based 2D and 3D image. The cerebral arteries ROIs were delineated and converted into color-coded 3D models using CTVox software.

### Statistical Analysis

SPSS 25.0 was used for statistical analysis. The significance of differences between two groups was determined by independent *t*-test if quantitative data were consistent with the normal distribution and homogeneity of variance. And results were presented as mean ± standard deviation of the mean (SEM). Rank-sum test was used for the comparison between groups if measurement data was non-normal distribution. *P* < 0.05 was considered statistically significant.

## Experimental Results

### Modeling Mortality Rate of Mice

In CG, the number of deaths was 1, 2, and 0 on the day of modeling, within the next 1–3 d, and during the recovery period of the next 14 d, respectively. And the overall mortality rate of mice was 10%. In EG, the number of deaths was 2, 3, 1, and 0 on the day of modeling, within the next 1–3 d, 3–7 d, and 7–14 d, respectively. And the overall mortality rate of mice was 20% (Table 1).

**Table 1:** Death records of mice in the two groups after modeling (*n* = 30).

**Group**	**Time (** ***d*** **)**				
	**<1**	**1 ≤*d* ≤3**	**3 < *d* ≤7**	**7 < *d* ≤14**	**0 < *d* ≤14**
Control group	1 (3.3%)	2 (6.7%)	0	0	3 (10%)
Experimental group	2 (6.7%)	3 (10%)	1 (3.3%)	0	6 (20%)

### Gross Anatomy

**Figure 2:**
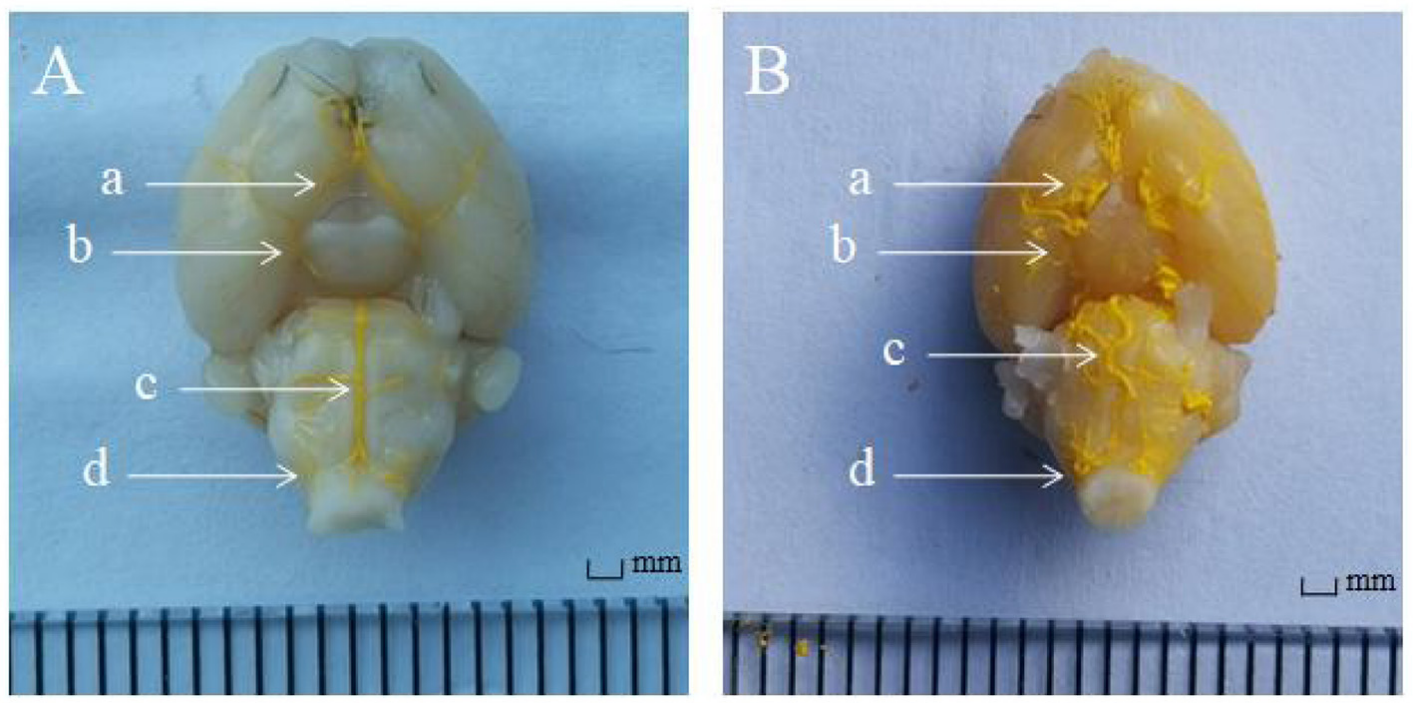
The angiography of FlowTech MICROFIL of intracranial vessels. **(A)** In the control group (*n* = 22), the intracranial vessels were smooth and normal. **(B)** In the experimental group (*n* = 19), the intracranial arteries were elongated and tortuous. Anterior cerebral artery A1 segment (a arrow), posterior communicating artery (b arrow), basilar artery (c arrow), and vertebral artery (d arrow).

After 2 weeks of injection of inactivated elastase, the A1 segment of the basilar artery, bilateral anterior cerebral arteries and bilateral posterior communicating arteries in CG was straight, without the performance of tortuosity, elongation, and dilation. Compared with CG, the A1 segment of the basilar artery, bilateral anterior cerebral arteries and bilateral posterior communicating arteries in EG was significantly tortuous, elongated, and dilated after 2 weeks injection of elastase (Figure 2).

### Morphological Measurement

Compared with CG, the basilar artery of was significantly tortuous, the actual length was notably increased, and the average elongation rate increased by 26% in EG. Meanwhile, the maximum diameter of the basilar artery, bilateral anterior cerebral arteries and bilateral posterior communicating arteries was markedly increased (*P* < 0.01, Tables 2, 3).

**Table 2:** Changes in curvature, actual length, and elongation rate of the basilar artery after modeling.

**Group**	**Bending angle (**°**)**	**Extended length (mm)**	**Average elongation rate (%)**
Control group (*n* = 22)	0	0	0
Experimental group (*n* = 19)	40 ± 5^a^	2.5 ± 0.21^a^	26%

*Compared with the control group*.

a *P < 0.01*.

**Table 3:** Changes in the maximum diameter (mm) of the basilar artery, anterior cerebral artery, and posterior communicating artery after elastase injection.

**Group**	**Basilar artery**	**Bilateral anterior cerebral arteries**	**Bilateral posterior communicating arteries**
Control group (*n* = 22)	0.19 ± 0.01	0.13 ± 0.01	0.15 ± 0.01
Experimental group (*n* = 19)	0.28 ± 0.01^a^	0.19 ± 0.02^a^	0.23 ± 0.02^a^

*Compared with the control group*.

a *P < 0.01*.

### Changes in Serum Inflammatory Factors

**Figure 3:**
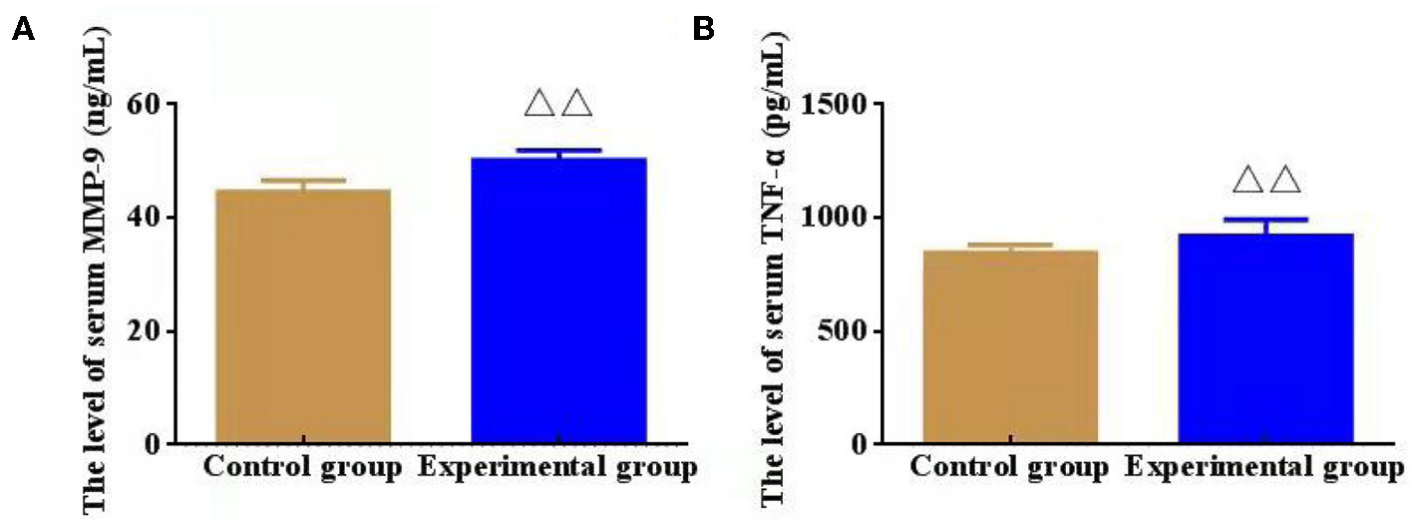
Effect of serum inflammatory factors, namely, matrix metalloproteinase (MMP-9) and lipoprotein (TNF-α, *n* = 8). **(A)** The level of serum MMP-9 (ng/mL); **(B)** The level of serum TNF-α (pg/mL). ^ΔΔ^*P* < 0.01 vs. Control group.

Compared with CG, the serum levels of inflammatory factors MMP-9 and TNF-α in EG were outstandingly increased (*P* < 0.01), which indicating that the obviously inflammatory reaction of intracranial vessels in mice after modeling (Figure 3).

### Histological Treatment and Staining of EVG

**Figure 4:**
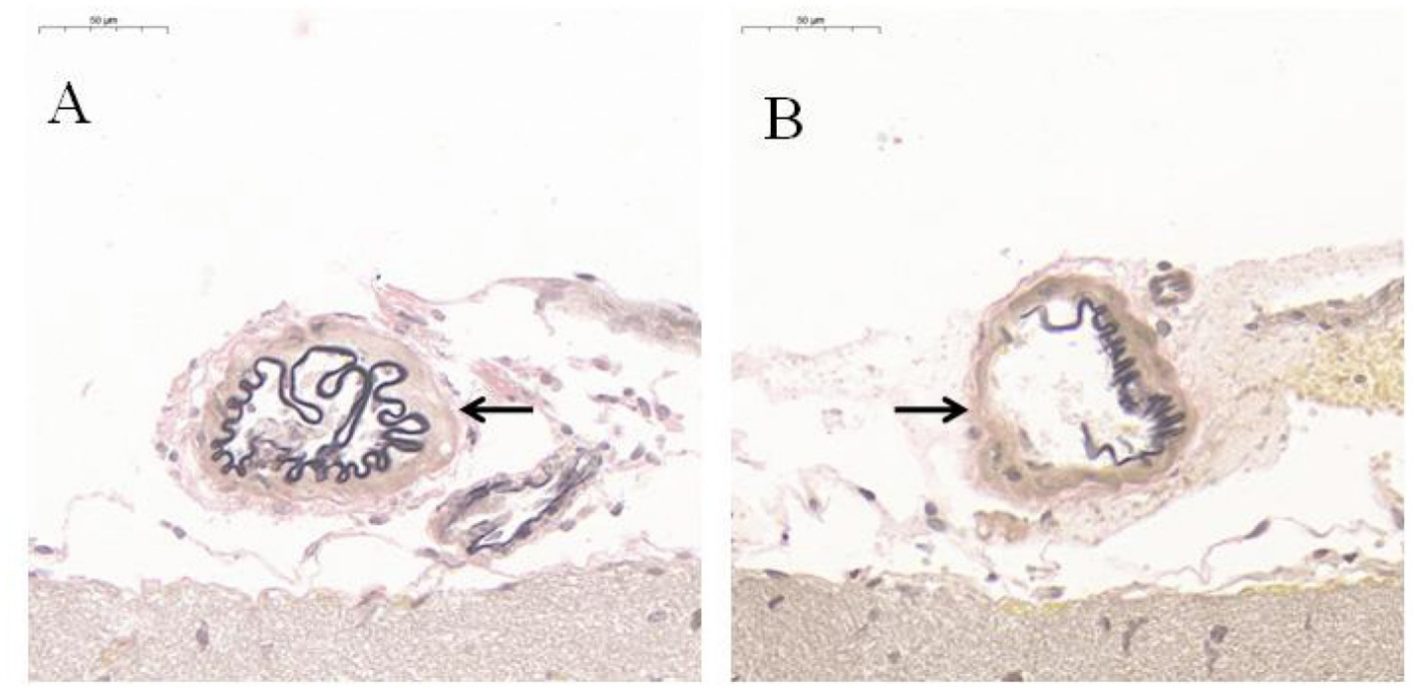
EVG histological staining of communicating artery vessels (*n* = 5). **(A)** Control group; **(B)** Experimental group. After modeling, the internal elastic lamina of the tunica media of the communicating artery was disrupted black arrow; the muscular layer was atrophied, and the connective tissue was hyalinized. However, no significant change was observed in the Control group black arrow.

Compared with CG, EVG stain showed that the destruction of the internal elastic layer, atrophy of the vascular muscles, and hyaline degeneration of connective tissue were observed in EG. There was no significant change observed in CG (Figure 4).

### Micro-CT Lamellar Scanning of Mice Cranial Arteries and Blood Vessels

**Figure 5:**
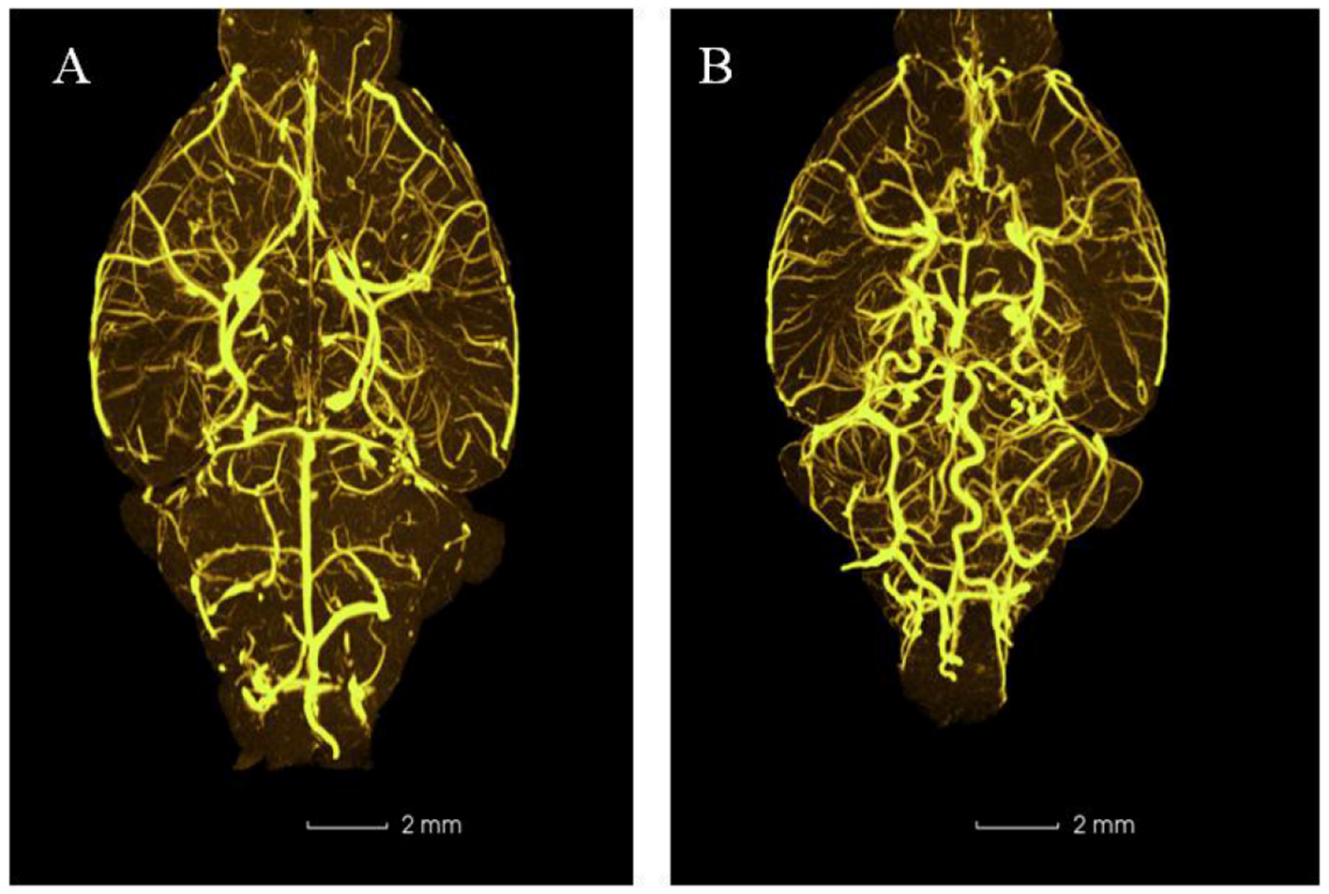
Representative three-dimensional images of mouse intracranial arteries (100×) (*n* = 5). **(A)** Control group; **(B)** Experimental group.

In EG, the A1 segments of an anterior cerebral artery, posterior communicating artery, basilar artery, and vertebral artery were obviously tortuous, elongated, and dilated after 2-week injection of elastase (Figure 5). There was no significant change in CG.

## Discussion

Not only does a mature animal model that accurately mimics human diseases contribute to elucidating the basic pathomechanism of the disease, but also it provides a good mock object for the disease diagnosis and treatment. Previous studies have proved that IADE, a progressive disease with unknown etiology and low detection rate, may have to do with congenital variations and fail to require special interventions. Nevertheless, the studies carried out in recent years have found an evident correlation between IADE and the occurrence and recurrence of posterior circulation ischemic stroke (12, 13). Therefore, the exploration of IADE animal models is needed desperately. In previous studies, there was a mere description of establishing process of the IADE mouse models roughly, and no detailed experimental protocol was offered. Additionally, the mortality rate of mice was higher, while the success rate of the model seemed lower (10, 11). By conducting a series of preliminary explorations, we had modified the previous research protocols and recorded the crucial links of the experiment and various experimental conditions in detail in order to provide a certain reference for related researchers.

Matrix metalloproteinases (MMP), as a type of zinc-binding proteolytic enzyme, is able to degrade extracellular matrix (ECM) proteins including elastase, collagen, and glycoprotein polymers. However, this degrading process may thin or destroy the internal elastic lamina of arteries (14). In previous studies, we found that a high level of plasma MMP-9 was associated with basilar arteriectasia in dizziness or vertigo patients (15, 16). Experiments on the elastase-induced IADE mouse model demonstrated that arterial wall dilation was characterized by internal elastic lamina rupture, inflammatory cell infiltration, and high expressions of MMP-2 and MMP-9 (10). TNF-α as a vital pro-inflammatory factor is mainly secreted by activating immune cells, which participates in immune regulation of the body. Also, TNF-α can be produced by vascular endothelial cells. High levels of TNF-α in vessels can cause adhesion and inflammation reaction of vascular endothelial cells, thereby damaging blood vessels and causing a variety of vascular diseases. In IADE development, the feedback loop between macrophages and inflammatory cytokines involves NF-kB signaling pathways and TNF-α and leads to subsequent ECM degeneration and MMP-9 activation (17). As a result, the suppression of macrophage-mediated inflammations and MMP activity could effectively prevent IADE formation. Our study found that the levels of MMP-9 and TNF-α in the peripheral blood of IADE mice were outstandingly increased, indicating the inflammatory reaction and vascular damage in intracranial arteries.

Experiment of elastinase injected into posterior cistern of mice is a more refined process. The survival rate and the successful rate of mice are determined by each step of modeling. Stereotactic injection is a useful technique to delivery medicine to targeted brain areas in mice, which could ensure that the injury to the brain is as minor as possible and repeated safely (18). A stereotactic frame was also previously used in the skull base injection, like the basal cistern. In the past, extracting cerebrospinal fluid through the cisterna magna or administering medicine often required special instruments and tools, for example, making a needle tip of the microsyringe into a hook shape or making an instrument in the lower part of the mouse body to fully expose the occipital cistern, which often caused some confusion to researchers (19, 20). Through many explorations, we have established the best parameters of the stereotaxic device, including maxillary fixator with a height of 14–15 mm, bilateral ear taper with a height of 15–16 mm, and bilateral ear taper with a width of 5–6 mm. Moreover, the mouse head is parallel to the plane, an angle of 110–120° is made with the body, and the ear taper is fixed in the pre-auricular temporal bone, then the position of the mouse cisterna magna can be fully exposed. At the moment, the field of view is adequate, which is conducive to needle insertion.

Related studies only described the status of mice within 24 h after surgery, and little attention was paid to the survival status of mice after 24 h (10, 11). To note the impact of surgery on animals, we recorded the mortality of mice within 24 h, 1–3 d, 4–7 d, and 8–14 d and the overall mortality after the surgery. We study found that most of the mice deaths after modeling were highly within 24 h and 3 d after the operation. Moreover, the mice were basically tolerant after 1 week of the operation and the mortality rate was reduced. Through the animal autopsy, the death of these mice was not only the physical damage to the brain stem and cerebellum caused by the microsyringe during the operation but also the cerebral angiolysis by elastase caused mice's intolerance. Our research shows that the mortality rate of mice within 14 d after modeling is 20%, which is 10% lower than the mortality reported in previous researches.

Furthermore, the depth of needle insertion proves the key factor indicating the success or failure of the experiment. By measuring the distance from the dura mater to the brainstem, we found that the average distance between them remains ~1.5 mm. Considering that the automatic injection pumps of the brain stereotaxic apparatus are in a vertical type and the lateral needle insertion apparatuses or self-adjustment devices are lacking, the needle must be inserted by hands. While using this technique, the hands of the operator must not shake to keep the needle from moving back and forth, thereby having the brain tissues of mice injure. The factor of handshaking during the needle insertion is also the primary reason for the high mortality of mice in our previous studies. To resolve this tricky problem, we have developed a modified needle-holding posture which can prevent handshaking and reduce the mice mortality rate of the operation.

In previous studies, polyvinylpyrrolidone was used to seal hermetically the pinhole, to avoid the outflow of elastase (21, 22). However, cerebrospinal fluid is easy to flow out with the action of intracranial pressure, as well as elastase solution. Therefore, a cotton swab was applied to press the pinhole immediately after the injection was completed, which may play an crucial role in the success rate of induction model. In this study, there were dolichoectasia and tortuosity of basilar artery, anterior cerebral artery, middle cerebral artery, and other blood vessels.

Elastase is able to degrade a variety of proteins, prevent cholesterol synthesis in the body, and promote its conversion to cholicacid. In recent years, elastase has been used to induce animal models such as intracranial aneurysm, cervical common aneurysm, abdominal aortic aneurysm in rabbits, emphysema, chronic obstructive pulmonary disease, and osteoporosis (23–28), which can obviously induce the pathological changes of the target blood vessels and tissues with the advantages of simple, convenient and high success rate. Elastase can be extracted and purified from the animal pancreas, which pertains to the endopeptidase and contains 240 amino acid residues with the isoelectric point of 9.5 (29) and the best pH value of 8.1–8.8. Its enzyme activity would be maintained for several days at 4–6°C with the pH value of 5–10, while its freeze-dried powder would be maintained for 6–12 months below 5°C (29). Given the instability of elastase in the normal atmospheric temperature, it is gradually inactivated with the temperature increase. However, it has not been accurately reported whether the enzyme will gradually inactivate and reduce its efficacy at a long-time indoor temperature. During the whole experiment, to ensure the effectiveness of enzyme activity, we prepared elastase when using and kept the prepared elastase at 0°C condition to ensure the effectiveness of enzyme activity, which may seem a principal factor indicating the high success rate of our elastase-induced model.

There may be some possible limitations in this study. First, First, the mortality of mice using the modeling protocol that we provided is still relatively high (20%). Therefore, it is necessary to explore a safer protocol to reduce the mortality of mice. Second, owing to hypertension also as a key factor indicating IADE occurrence, we should combine this modified protocol with the means of hypertension-induced model, so that it could be veritably consistent with the pathological characteristics of IADE. Third, the pathogenesis and signaling pathways of IADE *in vivo* need to be explored sufficiently.

In conclusion, by modifying and optimizing the modeling protocol, we established an animal IADE model that was with features of short time consumption, convenience and feasibility, low mortality rate and high success rate of modeling. This study may provide a mature and stable mouse model to contribute to exploring IADE internal mechanism and developing IADE-related drugs.

## Data Availability Statement

The raw data supporting the conclusions of this article will be made available by the authors, without undue reservation.

## Ethics Statement

The animal study was reviewed and approved by First Affiliated Hospital of Henan University of CM. Written informed consent was obtained from the owners for the participation of their animals in this study.

## Author Contributions

FL, YN, and RS conducted the experiment and wrote the first draft. DZ conceptualized the study. HZ, ZZ, and YZ revised the manuscript. All authors contributed to the article and approved the submitted version.

## Funding

The Special Project for Scientific Research of Traditional Chinese Medicine in Henan Province (Grant Nos. 2019ZY1013, 2019JDZX2101, and 2019JDZX2032), the National Natural Science Foundation of China (Grant No. 81573874), and Leading Talent Project of Henan Chinese Medicine Department (Yu Wei Zhong Yi Han [2021] No. 8) provided financial support for the conduct of the research.

## Conflict of Interest

The authors declare that the research was conducted in the absence of any commercial or financial relationships that could be construed as a potential conflict of interest.

## Publisher's Note

All claims expressed in this article are solely those of the authors and do not necessarily represent those of their affiliated organizations, or those of the publisher, the editors and the reviewers. Any product that may be evaluated in this article, or claim that may be made by its manufacturer, is not guaranteed or endorsed by the publisher.

## Abbreviations

IADE: intracranial arterial dolichoectasia
MMP-9: matrix metalloproteinase-9
TNF-α: tumor necrosis factor α.
